# Dissimilatory nitrate reduction by *Aspergillus terreus* isolated from the seasonal oxygen minimum zone in the Arabian Sea

**DOI:** 10.1186/1471-2180-14-35

**Published:** 2014-02-11

**Authors:** Peter Stief, Silvia Fuchs-Ocklenburg, Anja Kamp, Cathrine-Sumathi Manohar, Jos Houbraken, Teun Boekhout, Dirk de Beer, Thorsten Stoeck

**Affiliations:** 1 Max Planck Institute for Marine Microbiology, Microsensor Group, Bremen, Germany; 2 Department of Biology, University of Southern Denmark, NordCEE, Campusvej 55, 5230 Odense M, Denmark; 3 Department of Ecology, University of Kaiserslautern, Kaiserslautern, Germany; 4 Jacobs University Bremen, Molecular Life Science Research Center, Bremen, Germany; 5 CSIR-National Institute of Oceanography, Dona Paula, Goa, India; 6 CBS-KNAW Fungal Diversity Centre, Utrecht, The Netherlands; 7 Institute of Microbiology, Chinese Academy of Sciences, Beijing, China

## Abstract

**Background:**

A wealth of microbial eukaryotes is adapted to life in oxygen-deficient marine environments. Evidence is accumulating that some of these eukaryotes survive anoxia by employing dissimilatory nitrate reduction, a strategy that otherwise is widespread in prokaryotes. Here, we report on the anaerobic nitrate metabolism of the fungus *Aspergillus terreus* (isolate An-4) that was obtained from sediment in the seasonal oxygen minimum zone in the Arabian Sea, a globally important site of oceanic nitrogen loss and nitrous oxide emission.

**Results:**

Axenic incubations of An-4 in the presence and absence of oxygen and nitrate revealed that this fungal isolate is capable of dissimilatory nitrate reduction to ammonium under anoxic conditions. A ^15^N-labeling experiment proved that An-4 produced and excreted ammonium through nitrate reduction at a rate of up to 175 nmol ^15^NH_4_^+^ g^-1^ protein h^-1^. The products of dissimilatory nitrate reduction were ammonium (83%), nitrous oxide (15.5%), and nitrite (1.5%), while dinitrogen production was not observed. The process led to substantial cellular ATP production and biomass growth and also occurred when ammonium was added to suppress nitrate assimilation, stressing the dissimilatory nature of nitrate reduction. Interestingly, An-4 used intracellular nitrate stores (up to 6–8 μmol NO_3_^-^ g^-1^ protein) for dissimilatory nitrate reduction.

**Conclusions:**

Our findings expand the short list of microbial eukaryotes that store nitrate intracellularly and carry out dissimilatory nitrate reduction when oxygen is absent. In the currently spreading oxygen-deficient zones in the ocean, an as yet unexplored diversity of fungi may recycle nitrate to ammonium and nitrite, the substrates of the major nitrogen loss process *anaerobic ammonium oxidation*, and the potent greenhouse gas nitrous oxide.

## Background

In marine ecosystems, nitrate (NO_3_^-^) serves as both a nitrogen source for assimilation and an electron acceptor for dissimilatory processes when oxygen (O_2_) is deficient. The latter scenario is ubiquitously encountered in anoxic sediment layers, but also prevails in the water bodies of oxygen minimum zones (OMZs) of the world’s oceans [1]. In *denitrification*, nitrate is sequentially reduced to dinitrogen $\left({\mathrm{NO}}_{3}^{-}\to {\mathrm{NO}}_{2}^{-}\to \mathrm{NO}\to N_{2}O\to N_{2}\right)$, in *dissimilatory nitrate reduction to ammonium* (DNRA), nitrate is sequentially reduced to ammonium $\left({\mathrm{NO}}_{3}^{-}\to {\mathrm{NO}}_{2}^{-}\to {\mathrm{NH}}_{4}^{+}\right),$ and in *anaerobic ammonium oxidation* (anammox), ammonium is oxidized by nitrite to form dinitrogen $\left({\mathrm{NH}}_{4}^{+}+{\mathrm{NO}}_{2^{-}}\to N_{2}\right)$. These different metabolic pathways of dissimilatory ${\mathrm{NO}}_{3}^{-}$ or ${\mathrm{NO}}_{2}^{-}$ reduction were originally thought to only occur in prokaryotes [2-4]. Meanwhile, denitrification and DNRA have been discovered in a limited set of eukaryotic microorganisms, including marine foraminifers [5,6] and diatoms [7,8]. Incomplete denitrification to nitrous oxide (N_2_O) has also been proven for plant-pathogenic and soil fungi, such as *Fusarium oxysporum*[9,10], but so far not for marine isolates. Additionally, a large number of fungal species, mainly belonging to Ascomycota, are capable of “ammonia fermentation”, a form of ${\mathrm{NO}}_{3}^{-}$ reduction to ammonium $\left({\mathrm{NH}}_{4}^{+}\right)$ coupled to the fermentation of organic compounds [11].

Fungi are primarily aerobic heterotrophs, but some species, especially fermentative yeasts, can survive and grow under completely anoxic conditions. Nevertheless, both the abundance and the ecological role of fungi in O_2_-deficient marine environments are probably underestimated [12]. Recent sequencing approaches revealed a large diversity of marine microbial eukaryotes in environments where O_2_ occurs in low concentrations or is completely absent [13]. Additionally, it was found that fungal 18S rDNA sequences dominate the eukaryotic microbial communities in anoxic marine habitats (reviewed by [14]). Fungi retrieved from coastal marine sediments are dominated by Ascomycota that may be of terrestrial origin [15]. Amongst others, they are represented by *Aspergillus* species, including *A. terreus*[16]. Fungal community structures differ between oxic, seasonally anoxic, and permanently anoxic sites, suggesting adaptation of fungal communities to prevailing O_2_ conditions [12].

The Arabian Sea harbors two different O_2_-deficient conditions, which includes a seasonal OMZ along the continental shelf and an open-ocean, perennial OMZ [17]. The distribution of anaerobic nitrogen cycling in the Arabian Sea is patchy and covers areas with predominant denitrification [18] or anammox activity [19]. The Arabian Sea is also a globally important site of N_2_O emission [17,20,21]. The oversaturation of the water column with this potent greenhouse gas is ascribed to denitrification activity [17].

Here, the ecophysiology of an *A. terreus* isolate (An-4) obtained from the seasonal OMZ in the Arabian Sea was studied. An-4 was enriched from coastal sediment sampled during a period of bottom-water anoxia using anoxic, ${\mathrm{NO}}_{3}^{-}$-amended conditions. It was therefore hypothesized that An-4 is capable of dissimilatory NO_3_^-^ reduction. The role of O_2_ and ${\mathrm{NO}}_{3}^{-}$ availability in triggering dissimilatory NO_3_^-^ reduction was studied in axenic incubations. In a dedicated ^15^N-labeling experiment, all environmentally relevant products of dissimilatory ${\mathrm{NO}}_{3}^{-}$ reduction were determined. Intracellular ${\mathrm{NO}}_{3}^{-}$ storage, a common trait of NO_3_^-^-respiring eukaryotes, was studied combining freeze-thaw cycles and ultrasonication for lysing ${\mathrm{NO}}_{3}^{-}$-storing cells. Production of cellular energy and biomass enabled by dissimilatory ${\mathrm{NO}}_{3}^{-}$ reduction was assessed with ATP and protein measurements, respectively. Using these experimental strategies, we present the first evidence for dissimilatory ${\mathrm{NO}}_{3}^{-}$ reduction by an ascomycete fungus that is known from a broad range of habitats, but here was isolated from a marine environment.

## Results

### Aerobic and anaerobic nitrate and ammonium turnover

**Figure 1:**
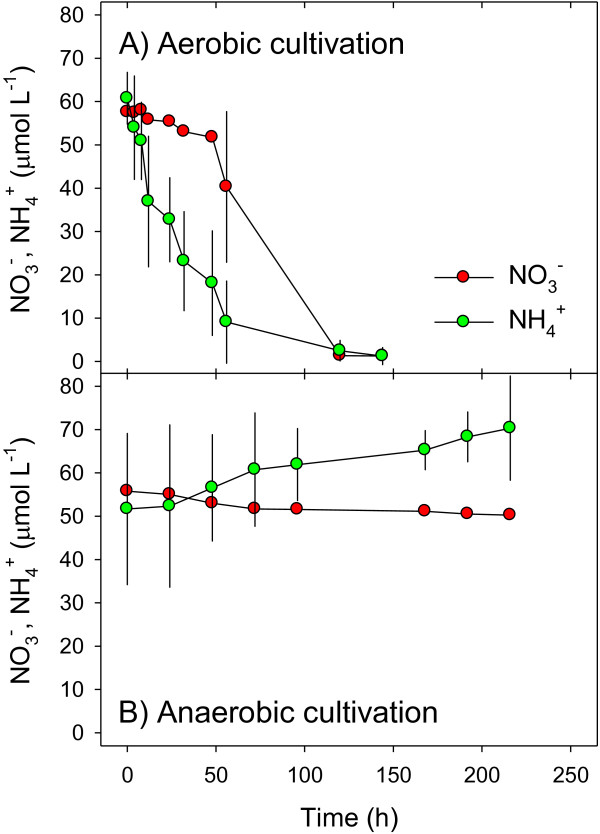
**Time course of nitrate and ammonium concentrations during axenic cultivation of *A. terreus *isolate An-4 (Experiment 1). (A)** Aerobic, **(B)** anaerobic cultivation. The liquid media were amended with nominally 50 μmol L^-1^ of NO_3_^-^ and NH_4_^+^ each at the beginning of cultivation. Means ± standard deviation (n = 3).

**Figure 2:**
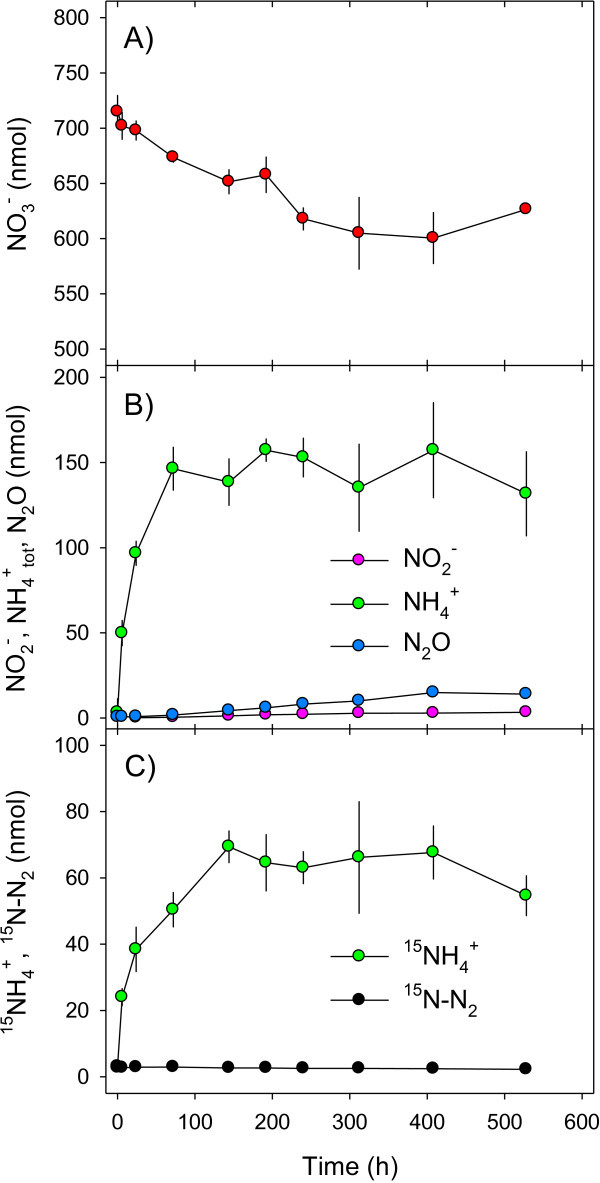
**Time course of inorganic nitrogen species during anaerobic incubation of *A. terreus *isolate An-4 (Experiment 2).** The isolate was pre-cultivated under oxic conditions with ^15^NO_3_^-^ as the only source of NO_3_^-^ and then exposed to anoxic conditions. Absolute amounts of **(A) **^15^N-labeled NO_3_^-^, **(B)** total NO_2_^-^, total NH_4_^+^, and total N_2_O, and **(C) **^15^N-labeled NH_4_^+^ and N_2_ in the incubation vials are shown. Means ± standard deviation (n = 3).

**Figure 3:**
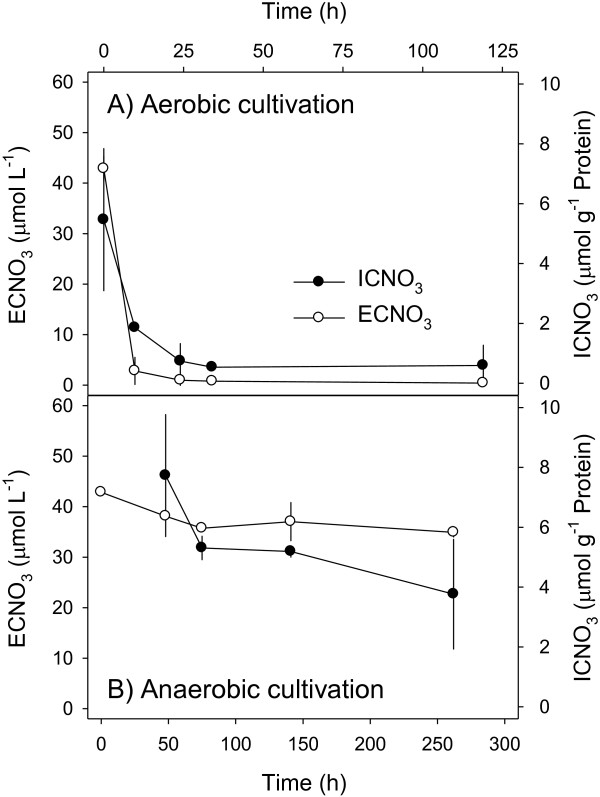
**Time course of intracellular nitrate contents (ICNO**_**3**_**) and extracellular nitrate concentrations (ECNO**_**3**_**) (Experiment 3)**. *A. terreus* isolate An-4 was cultivated under **(A)** oxic and **(B)** anoxic conditions. ICNO_3_ contents are expressed per g protein of the fungal biomass. Means ± standard deviation (n = 3).

The fate of ${\mathrm{NO}}_{3}^{-}$ added to the liquid media of axenic An-4 cultures (verified by microscopy and PCR screening, see Methods) was followed during aerobic and anaerobic cultivation (Experiment 1), in a ^15^N-labeling experiment involving an oxic-anoxic shift (Experiment 2), and in a cultivation experiment that addressed the intracellular storage of ${\mathrm{NO}}_{3}^{-}$ (Experiment 3). Nitrate was generally consumed, irrespective of O_2_ availability (Figures 1A + B (Exp. 1), 2A (Exp. 2), and 3A + B (Exp. 3)). Under oxic conditions, ${\mathrm{NO}}_{3}^{-}$ concentrations in the liquid media exhibited sudden drops when high biomass production and/or ${\mathrm{NH}}_{4}^{+}$ depletion was noted in the culture flasks (Figures 1A and 3A). Under anoxic conditions, however, ${\mathrm{NO}}_{3}^{-}$ concentrations in the liquid media decreased steadily over the whole incubation period during which neither sudden increases in biomass production, nor ${\mathrm{NH}}_{4}^{+}$depletion were noted (Figures 1B, 2A, and 3B).

**Figure 4:**
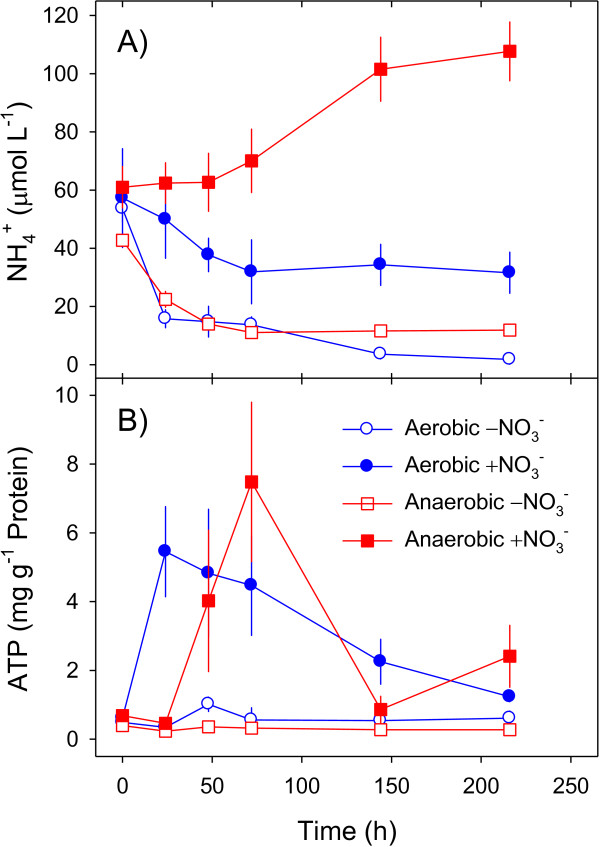
**Time course of extracellular ammonium concentrations and adenosine triphosphate (ATP) contents of *A. terreus *isolate An-4 (Experiment 4). (A)** Ammonium concentrations in the liquid media and **(B)** biomass-specific ATP contents of *A. terreus* isolate An-4 were determined during aerobic and anaerobic cultivation in the presence or absence of NO_3_^-^. ATP contents are expressed per g of protein of the fungal biomass. Means ± standard deviation (n = 3).

The fate of ${\mathrm{NH}}_{4}^{+}$ was investigated in Experiments 1 and 2 and additionally in an experiment that addressed the production of biomass and cellular energy during aerobic and anaerobic cultivation (Experiment 4). Ammonium was either net consumed or net produced, which depended on the availability of both O_2_ and ${\mathrm{NO}}_{3}^{-}$ (Figures 1A + B, 2B + C, and 4A (Exp. 4)). In the absence of ${\mathrm{NO}}_{3^{-}},\;{\mathrm{NH}}_{4^{+}}$ was invariably consumed, irrespective of O_2_ availability (Figure 4A). In the presence of ${\mathrm{NO}}_{3}^{-}$, ${\mathrm{NH}}_{4}^{+}$ was either consumed or produced under oxic and anoxic conditions, respectively (Figures 1A + B, 2B + C, and 4A). Taken together, these results suggest a role of ${\mathrm{NO}}_{3}^{-}$ in nitrogen assimilation under oxic conditions when ${\mathrm{NH}}_{4}^{+}$ is depleted, and a role of NO_3_^-^ in dissimilation under anoxic conditions when ${\mathrm{NH}}_{4}^{+}$is available. Additionally, the net production of NH_4_^+^ under anoxic conditions suggests dissimilatory ${\mathrm{NO}}_{3}^{-}$reduction to ${\mathrm{NH}}_{4}^{+}$ by An-4.

### Products of anaerobic nitrate turnover

The precursors, intermediates, and end products of dissimilatory NO_3_^-^ reduction (i.e., NO_3_^-^, NO_2_^-^, NH_4_^+^, N_2_O, and N_2_) by An-4 were investigated in a ^15^N-labeling experiment (Exp. 2). Axenic mycelia were incubated with ^15^NO_3_^-^ and then subjected to a sudden oxic-anoxic shift. The anaerobic consumption of NO_3_^-^ by An-4 was accompanied by the production and cellular release of NH_4_^+^, NO_2_^-^, and N_2_O, but not N_2_ (Figure 2A-C). Ammonium was quantitatively by far the most important product, whereas N_2_O and NO_2_^-^ were less important (Figure 2B + C, Table 1, Additional file 1: Figure S1). Biomass-specific ^15^NH_4_^+^ production rates equaled ^15^NO_3_^-^ consumption rates during the first 3 days of incubation (Table 1). During the remaining incubation time, N consumption and production rates were generally lower than during the first 3 days (Table 1). After no further decrease of the NO_3_^-^ concentration was observed (i.e., after 408 h), NH_4_^+^, N_2_O, and NO_2_^-^ formed 83.0, 15.5, and 1.5%, respectively, of all N produced and released into the liquid media. These results substantiate the capability of An-4 to dissimilatorily reduce NO_3_^-^ to NH_4_^+^ (as main product), NO_2_^-^ and N_2_O (as side products) under anoxic conditions.

**Table 1:** **Turnover rates of inorganic nitrogen species by *A. terreus *isolate An-4 during anaerobic incubation with **^**15**^**NO**_**3**_^**- **^**enrichment (Experiment 2)**

**Nitrogen species**	** Day 0-3**	** Day 3-17**
NO_3_^-^_total_	−166.5 (33.9)	−76.4 (13.3)
NO_2_^-^_total_	+3.4 (0.4)	+1.5 (0.3)
NH_4_^+^_total_	+565.4 (74.8)	+6.1 (12.4)
N_2_O_total_	+5.0 (0.7)	+12.5 (0.9)
^15^NH_4_^+^	+175.4 (33.7)	+11.1 (6.5)
^15^N-N_2_	+0.7 (0.8)	−0.4 (0.2)

Rates were calculated for linear increases or decreases in the amount of the different nitrogen species during the early and late phase of anaerobic incubation. Mean rates (standard error) are given as nmol N g^-1^ protein h^-1^. Positive and negative values indicate production and consumption, respectively.

### Intracellular nitrate storage

The capability of An-4 to store nitrate intracellularly, a common trait of large-celled microorganisms that respire nitrate, was investigated during both aerobic and anaerobic cultivation (Exp. 3). Intracellular NO_3_^-^ concentrations (ICNO_3_) were high when extracellular NO_3_^-^ concentrations (ECNO_3_) were high and vice versa, irrespective of O_2_ availability (Figure 3A + B). Under oxic conditions, however, ICNO_3_ and ECNO_3_ concentrations dropped sharply within the first day of incubation (Figure 3A), whereas under anoxic conditions, steady decreases in ICNO_3_ and ECNO_3_ concentrations were noted during 11 days of incubation (Figure 3B).

In the ^15^N-labeling experiment (Exp. 2), the total amount of N produced in each incubation vial (185.4 ± 29.3 nmol) exceeded the total amount of NO_3_^-^ consumed (114.4 ± 27.3 nmol), implying that also 71.0 nmol ICNO_3_ was consumed during the anoxic incubation. The initial amount of ICNO_3_ transferred into the incubation vials together with the An-4 mycelia of 77.5 ± 28.9 nmol equaled the calculated amount of ICNO_3_ needed to close the N budget.

### Production of biomass and cellular energy

The production of biomass and cellular energy by An-4 was studied during aerobic and anaerobic cultivation in the presence or absence of NO_3_^-^ (Experiment 4); biomass production was also recorded in Experiment 1. For this purpose, the time courses of protein and ATP contents of An-4 mycelia and of NO_3_^-^ and NH_4_^+^ concentrations in the liquid media were followed. Biomass production by An-4 was significantly higher when O_2_ and/or NO_3_^-^ were available in the liquid media (Table 2). The biomass-specific ATP contents of An-4 reached higher values when NO_3_^-^ was available in the liquid media and were invariably low in its absence (Figure 4B). Under oxic conditions, ATP contents increased to maximum values within 1 day of incubation and steadily decreased during the following 8 days (Figure 4B). Under anoxic conditions, ATP contents reached maximum values only after 3 days and thereafter fluctuated around intermediate values (Figure 4B). These results substantiate the capability of An-4 to grow anaerobically and produce cellular energy by dissimilatory NO_3_^-^ reduction to NH_4_^+^.

**Table 2:** **Correlation between oxygen and nitrate availability and biomass production by *A. terreus *isolate An-4 (Experiments 1 and 4)**

**Experiment**	**Treatment**	**Nitrate in media (μM)**	**Final biomass in flask (g)**
Experiment 1	Aerobic + Nitrate	43.2 (1.7)	11.4 (1.5)
	Anaerobic + Nitrate	52.3 (0.5)	1.5 (0.1)
Experiment 4	Aerobic – Nitrate	3.4 (0.1)	2.2 (0.4)
	Aerobic + Nitrate	30.6 (2.7)	11.2 (1.0)
	Anaerobic – Nitrate	6.6 (0.1)	0.7 (0.1)
	Anaerobic + Nitrate	95.4 (8.7)	2.3 (1.8)

Nitrate concentrations are given as the mean (standard deviation) of 6–10 samples taken during the cultivation period. Final biomass is given as the mean (standard deviation) wet weight of three fungal cultures harvested at the end of the cultivation period. The final biomass does not include the (minor) weight of six samples that were taken for protein and ATP analysis in Experiment 4.

## Discussion

### Physiology of isolate An-4

All observations made during incubations of *Aspergillus terreus* (isolate An-4) in the presence and absence of O_2_ and NO_3_^-^ indicate that this fungus is capable of dissimilatory NO_3_^-^ reduction to NH_4_^+^[11]. An-4 produced NH_4_^+^ only under anoxic conditions and through NO_3_^-^ reduction as proven in the ^15^N-labeling experiment. The process led to significant cellular ATP production and biomass growth and also occurred when NH_4_^+^ was added to suppress NO_3_^-^ assimilation, stressing the dissimilatory nature of the observed anaerobic NO_3_^-^ reduction activity. For a large number of other fungal species, this type of anaerobic NO_3_^-^ metabolism has been termed “ammonia fermentation” in case that the reduction of NO_3_^-^ to NH_4_^+^ was coupled to the oxidation of organic carbon compounds to acetate and substrate-level phosphorylation [10,11]. Ammonia fermentation has been found in a wide spectrum of filamentous ascomycetous fungi [11,22], but so far not in fungi isolated from marine environments. Since the fermentation of organic substrates is not proven for An-4, the anaerobic NO_3_^-^ metabolism of this isolate might as well be of respiratory nature and then corresponds to DNRA. This pathway has so far been excluded to occur in fungi because a pentaheme cytochrome *c* NO_2_^-^ reductase typical of DNRA [23] has not been found in fungi with an anaerobic NO_3_^-^ metabolism [24].

Aside from the general accord with fungal ammonia fermentation or DNRA, the anaerobic NO_3_^-^ metabolism of An-4 showed several interesting features. Most notably, dissimilatory NO_3_^-^ reduction was accompanied by significant N_2_O production (ca. 15% of NO_3_^-^ reduced) and to a lesser extent by NO_2_^-^ production (ca. 1.5% of NO_3_^-^ reduced). While it was not surprising that traces of NO_2_^-^, an intermediate of dissimilatory NO_3_^-^ reduction to NH_4_^+^, were released into the liquid media [8,11], the production and cellular release of N_2_O was unexpected [10]. Nitrous oxide is the end product of incomplete denitrification in many plant-pathogenic and soil fungi [9,25,26], whereas the marine isolate An-4 obviously produces N_2_O via dissimilatory NO_3_^-^ reduction to NH_4_^+^. Nitrous oxide is not generally known as an *intermediate* of dissimilatory NO_3_^-^ reduction to NH_4_^+^, but may well be a *by-product* of this reduction pathway as shown for bacteria [27-29].

An-4 is clearly able to store NO_3_^-^ intracellularly and use it for dissimilatory NO_3_^-^ reduction to NH_4_^+^. Intracellular NO_3_^-^ storage is known for a number of prokaryotic and eukaryotic microorganisms capable of dissimilatory NO_3_^-^ reduction, but so far has not been reported for fungi, even when capable of denitrification or ammonia fermentation [10,24]. Large sulfide-oxidizing bacteria [30,31], foraminifers and gromiids [5,6,32,33], and diatoms [7,8,34,35] store NO_3_^-^ in their cells in millimolar concentrations. In our experiments with An-4, the maximum biomass-specific intracellular NO_3_^-^ contents were 6–8 μmol g^-1^ protein. Assuming a cellular protein content of 50% of the dry weight and a cellular water content of 90% of the wet weight, maximum intracellular nitrate concentrations reached ca. 400 μmol L^-1^. This intracellular NO_3_^-^ pool proved to be quantitatively important for dissimilatory NO_3_^-^ reduction by An-4, since it contributed up to 38% to the total NO_3_^-^ consumption in the ^15^N-labeling experiment. The initially high rates of NH_4_^+^ production may suggest that An-4 is first using up the readily available intracellular NO_3_^-^ stores before it switches to using extracellular NO_3_^-^ as well, but this scenario needs to be proven in a dedicated ^15^N-labeling experiment. The general physiology of intracellular NO_3_^-^ storage by An-4 is currently unknown. For instance, it is not clear at which growth stage and under which ambient conditions An-4 is taking up NO_3_^-^ from the environment because the phase of increasing intracellular NO_3_^-^ contents was not captured by our oxic and anoxic incubations. From the observed correlation between ICNO_3_ and ECNO_3_ it can be concluded that an unknown enrichment factor cannot be exceeded, meaning that ICNO_3_ concentrations will increase with ECNO_3_ concentrations, probably up to an as yet unknown maximum ICNO_3_ concentration. Benthic microorganisms that store NO_3_^-^ often show vertical migration behavior in the sediment that may enable them to take up NO_3_^-^ closer to the sediment surface and in the presence of O_2_[30,36,37]. It is conceivable that the hyphae of An-4 grow in direction of NO_3_^-^-containing layers closer to the sediment surface to facilitate NO_3_^-^ uptake. Finally, it remains to be investigated whether An-4 accumulates NO_3_^-^ in acidic vacuoles as recently shown for large sulfur bacteria [38] or in the cytosol of the hyphae.

### Ecological implications of anaerobic nitrate turnover by isolate An-4

*Aspergillus terreus* is a common and globally occurring soil fungus that is also known from substrates as diverse as air, salterns, capybara droppings, lung of pocket mice, corn, cotton plants, milled rice, muesli, and wall paint [39]. The species has been reported from marine and associated habitats, such as mangroves and soft corals, and isolates from these habitats have been widely investigated for the production of bioactive compounds [40-42]. *A. terreus* has also been isolated from the hypersaline water of the Dead Sea [43,44]. The species is an important human pathogen causing bronchopulmonary aspergillosis and disseminated infections [45]. Dissimilatory NO_3_^-^ reduction by human-associated microorganisms has been demonstrated [46,47], but it is not known whether fungi are involved. *A. terreus* is also of considerable biotechnological interest because it produces a wide diversity of secondary metabolites that find pharmaceutical applications, biotechnologically relevant compounds such as itaconic acid and itatartaric acid, as well as mycotoxins that are important for food safety ( [39] and references therein).

The wide habitat spectrum of *A. terreus* might be significantly expanded by the ability for dissimilatory NO_3_^-^ reduction in the absence of O_2_. This fungus has the potential to survive hypoxic or anoxic conditions that prevail in aquatic sediments mostly just a few millimeters below the surface [48] or even directly at the surface when O_2_ concentrations are low in the water column [12,49]. In contrast, NO_3_^-^ originating from the water column and/or the nitrification layer at the sediment surface diffuses deeper into the sediment than O_2_ does [50]. In shallow sediments, NO_3_^-^-rich water is introduced into even deeper layers by mixing forces such as bioturbation, bioirrigation, and ripple movement [51,52]. The sediment habitat in which *A. terreus* can thrive is further expanded by its NO_3_^-^ storage capability. The maximum intracellular NO_3_^-^ content of 8 μmol g^-1^ protein theoretically sustains dissimilatory NO_3_^-^ reduction without extracellular NO_3_^-^ supply for 2–4.5 days (calculated from rates measured in the ^15^N-labeling experiment). Survival and growth beyond this time frame will depend on the ability of *A. terreus* to repeatedly access NO_3_^-^ in its natural sediment habitat, which is currently unknown.

The dissimilatory NO_3_^-^ reduction activity of An-4 leads to the production and release of NH_4_^+^, N_2_O, and NO_2_^-^. Thus, unlike the denitrification and anammox activities of other microorganisms, the anaerobic NO_3_^-^ metabolism of An-4 cannot directly lead to fixed nitrogen removal. Since the major product of NO_3_^-^ reduction is NH_4_^+^, An-4 merely converts one form of fixed nitrogen into another one. It is noteworthy, however, that the production of NH_4_^+^ and NO_2_^-^ by An-4 might indirectly contribute to fixed nitrogen removal by fueling anammox, the dominant nitrogen loss process in many OMZs [53]. Remarkably, An-4 produces and releases ca. 15% of the total NO_3_^-^ reduced as N_2_O, a potent greenhouse gas [54,55]. Interestingly, the OMZs of the Arabian Sea have repeatedly been reported to be major sites of N_2_O production, especially in continental shelf areas and coastal upwelling zones [17,20,21,56].

## Conclusion

Before meaningful conclusions on the potential impact of fungi on the marine nitrogen cycle can be drawn, it has to be established how abundant and widespread fungi with an anaerobic NO_3_^-^ metabolism are in marine environments. Previous studies reported a high diversity of fungi in O_2_-deficient marine environments [12,16], a large proportion of which may have similar physiologies as An-4. Therefore, further concerted efforts should aim at revealing the so far largely ignored influence of fungi on the marine nitrogen cycle and their role in the production of greenhouse gases.

## Methods

### Geographic origin and identity of isolate An-4

The sampling site was located in the coastal, seasonal OMZ off Goa (India), northwest of the river mouths of the Zuari and the Mandovi (15°31′80″N, 73°42′60″E). Sampling was carried out at 14 m water depth in October 2005 and anoxic conditions were recorded in the bottom waters during sampling. Four ascomycete fungi were successfully isolated by the particle-plating technique after enrichment in anoxic, nitrate-amended seawater. One of the ascomycete isolates (An-4) was axenized with antibiotics and is tested here for its capability to reduce nitrate in the absence of oxygen.

Isolate An-4 was identified as *Aspergillus terreus* (Order Eurotiales, Class Eurotiomycetes) using morphological and DNA sequence data. Macro- and microscopic characters were studied according to [39]. Partial calmodulin (Cmd) and β-tubulin (BenA) gene sequences retrieved from the isolate with previously described methods [57,58] were used to derive the phylogenetic position of An-4 (Additional file 1: Figure S2). The obtained sequences were deposited in the NCBI GenBank sequence database under accession numbers [KJ146014] (Cmd) and [KJ146013] (BenA). The isolate was deposited in the culture collection of the CBS-KNAW Fungal Biodiversity Centre as [CBS 136781] and at the Microbial Type Culture Collection and Gene Bank (MTCC, Chandigarh, India) as [MTCC 11865].

### Cultivation for anaerobic nitrate turnover experiments

An-4 was pre-grown on agar plates prepared from YMG broth (i.e., Yeast extract [8 g L^-1^] + Malt extract [10 g L^-1^] + Glucose [10 g L^-1^]) supplemented with penicillin and streptomycin. Every few plate transfers, the antibiotics were omitted to avoid emergence and carry-over of resistant bacteria. Spores of the axenic isolate grown on agar plates were used to inoculate 500-mL Erlenmeyer flasks that contained 250 mL of YMG broth. For aerobic cultivation, the flasks were closed with aseptic cotton plugs. The flasks were placed on a rotary shaker (120 rpm) and incubated at 26°C. Under these conditions, the mycelia of An-4 formed spherical aggregates of 2–5 mm in diameter. The transfers from plate to flask were repeated every 3–4 weeks.

### Anaerobic nitrate turnover

The capability of An-4 to reduce nitrate anaerobically was investigated in two experiments: (1) An-4 was cultivated in Erlenmeyer flasks under oxic vs. anoxic conditions in the presence of both NO_3_^-^ and NH_4_^+^, and (2) An-4 was pre-cultivated in Erlenmeyer flasks under oxic conditions in the presence of ^15^NO_3_^-^ and then exposed to anoxic conditions in gas-tight incubation vials.

In Experiment 1, the fate of NO_3_^-^ and NH_4_^+^ added to the liquid media was followed during aerobic and anaerobic cultivation of An-4. Six replicate liquid cultures were prepared as described above, but with the YMG broth adjusted to nominal concentrations of 50 μmol L^-1^ NO_3_^-^ and 50 μmol L^-1^ NH_4_^+^ using aseptic NaNO_3_ and NH_4_Cl stock solutions, respectively. Three cultures were incubated aerobically, whereas the other three cultures were incubated anaerobically by flushing the Erlenmeyer flasks with dinitrogen for 30 min and then closing them with butyl rubber stoppers. Subsamples of the liquid media (1.5 mL) were taken after defined time intervals using aseptic techniques. Anaerobic cultures were sampled in an argon-flushed glove box to avoid intrusion of O_2_ into the Erlenmeyer flasks. Samples were immediately frozen at −20°C for later analysis of NO_3_^-^ and NH_4_^+^ concentrations.

In Experiment 2, the precursors, intermediates, and end products of dissimilatory nitrate reduction by An-4 were investigated in a ^15^N-labeling experiment, involving an oxic-anoxic shift imposed on axenic mycelia. For the aerobic pre-cultivation, a liquid culture was prepared as described above, but with the YMG broth adjusted to 120 μmol L^-1^^15^NO_3_^-^ (98 atom% ^15^N; Sigma-Aldrich). For anaerobic incubation, fungal aggregates were transferred to gas-tight glass vials (5.9-mL exetainers; Labco, Wycombe, UK) filled with anoxic NaCl solution (2%) amended with nitrate as electron acceptor and glucose as electron donor. Using aseptic techniques, equally-sized subsamples of fungal aggregates were transferred from the aerobic pre-cultures into 30 replicate exetainers. The wet weight of the aggregates was determined. Then the exetainers were filled with anoxic NaCl solution adjusted to 120 μmol L^-1^^15^NO_3_^-^ and 25 μmol L^-1^ glucose. Care was taken not to entrap any gas bubbles when the exetainers were closed with the septum cap. The exetainers were fixed in a rack that was continuously rotated to keep the aggregates in suspension and were incubated at 26°C in the dark for 24 days.

The anaerobic incubation was terminated in batches of three exetainers after defined time intervals. Subsamples of the liquid media were withdrawn through the septum (and simultaneously replaced with helium) for analyzing the concentrations of extracellular NO_3_^-^, NO_2_^-^, NH_4_^+^_total_, ^15^NH_4_^+^, and N_2_O, while the concentrations of ^15^N-N_2_O and ^15^N-N_2_ were determined directly in the incubation exetainers. For NO_3_^-^, NO_2_^-^, and NH_4_^+^_total_ analysis, 1.5 mL of the liquid media was immediately frozen at −20°C. For N_2_O analysis, 1 mL of the liquid media was immediately transferred into an N_2_-purged 3-mL exetainer and fixed with 100 μL ZnCl_2_ (50%). For ^15^NH_4_^+^ analysis, 0.5 mL of the liquid media was transferred into a 3-mL exetainer and frozen at −20°C. The liquid media remaining in the incubation exetainers were fixed with 100 μL ZnCl_2_ (50%) for later ^15^N-N_2_O and ^15^N-N_2_ analysis. For technical reasons, ^15^N-N_2_O could not be quantified for this specific experiment, but only for a slightly modified twin experiment the results of which are presented in the *Supporting Information*.

Additional exetainers with fungal aggregates were prepared and treated in the same way as the other exetainers for verifying that An-4 remained axenic throughout the anaerobic incubation. At the end of the experiment, these exetainers were opened using aseptic techniques and subsamples of both fungal aggregates (at least two) and liquid medium (100 μL) were plated on YMG agar. After incubation at 26°C for 15 days, the fungal colonies were carefully checked by microscopy for the presence of bacteria and xenic fungi. All microscopic checks were negative. Additionally, DNA was extracted from fungal aggregates and liquid medium with the UltraClean™ Soil DNA Isolation Kit (Mo Bio, Carlsbad, CA) and used as template for PCR targeting the 16S rRNA gene with the universal bacterial primers GM3F/GM4R [59]. All molecular checks were negative, since agarose gel electrophoresis did not reveal any specific amplification product except for in the positive control, a laboratory strain of *Agrobacterium* sp.

### Intracellular nitrate storage

The capability of An-4 to store nitrate intracellularly was investigated during both aerobic and anaerobic cultivation (Experiment 3). Liquid cultures were prepared as described above, but with the YMG broth adjusted to 50 μmol L^-1^ NO_3_^-^. After defined time intervals, YMG broth and fungal aggregates were subsampled for analysis of NO_3_^-^ freely dissolved in the broth (i.e., extracellular nitrate = ECNO_3_) and NO_3_^-^ contained within the fungal hyphae (i.e., intracellular nitrate = ICNO_3_). Subsamples for ECNO_3_ analysis (1.5 mL) were cleared from suspended hyphae by mild centrifugation at 1000× g for 10 min and the supernatants (S_0_) were stored at −20°C for later analysis. Fungal aggregates for ICNO_3_ analysis were collected in a 2-mL centrifugation tube and the adhering YMG broth was siphoned off using a hypodermic needle. The aggregates were washed with 1 mL nitrate-free NaCl solution (2%) and blotted dry on nitrate-free filter paper. The aggregates were then equally distributed among two 15-mL centrifugation tubes, one for ICNO_3_ analysis and one for protein analysis.

Aggregates intended for ICNO_3_ analysis were weighed and thoroughly mixed with 2.5 mL nitrate-free NaCl solution (2%) and centrifuged at 1000× g for 5 min. Half a milliliter of the supernatant (S_1_) was stored at −20°C for later analysis. To make the fungal hyphae burst and release the ICNO_3_ into the NaCl solution, the tube was alternately cooled down to −196°C in liquid nitrogen and heated up to +90°C in a water bath for 5 min each. Cell disruption was additionally promoted by a 1-min treatment with an ultrasonic probe (UW70, Bandelin, Germany). The homogenized hyphae were pelleted by centrifugation at 3000× g for 10 min and the supernatant (S_2_) was stored at −20°C for later analysis.

Aggregates intended for protein analysis were suspended in 4 mL 0.5 M NaOH, sonicated for 1 min, and incubated at +90°C for 15 min for hot alkaline extraction of cellular proteins. The hyphae were pelleted by centrifugation at 3000× g for 5 min and the supernatant was stored at −20°C for later protein analysis according to [60]. Protein extraction was repeated with the pelleted hyphae and the results of the analysis of the two supernatants were combined. A conversion factor (wet weight → protein content) was derived and used for calculating the biomass-specific ICNO_3_ contents as the difference between NO_3_^-^ concentrations in S_1_ and S_2_ divided by the protein contents of the hyphae.

### Production of biomass and cellular energy

The production of biomass and cellular energy by An-4 was studied during aerobic and anaerobic cultivation in the presence or absence of NO_3_^-^ (Experiment 4). For this purpose, the time courses of protein and ATP contents of An-4 mycelia and of NO_3_^-^ and NH_4_^+^ concentrations in the liquid media were followed. Twelve replicate liquid cultures were prepared as described for Experiment 1, but in six cultures NO_3_^-^ addition was omitted. Six cultures (3 cultures each with and without NO_3_^-^) were incubated aerobically, whereas the other six cultures (3 cultures each with and without NO_3_^-^) were incubated anaerobically. Subsamples of the liquid media (1.5 mL) and An-4 mycelia (4–6 aggregates) were taken after defined time intervals using aseptic techniques. Samples were immediately frozen at −20°C for later analysis of NO_3_^-^ and NH_4_^+^ concentrations and protein and ATP contents. The NO_3_^-^-amended cultures received additional NO_3_^-^ (to a nominal concentration of 50 μmol L^-1^) after 1, 3, 7, and 9 days of incubation to avoid premature nitrate depletion.

### Nitrogen analyses

Nitrate and NO_2_^-^ were analyzed with the VCl_3_ and NaI reduction assay, respectively [61,62]. In these methods, NO_3_^-^ and/or NO_2_^-^ are reduced to nitric oxide that is quantified with the chemiluminescence detector of an NO_x_ analyzer (CLD 60, Eco Physics, Munich, Germany). Ammonium was analyzed with the salicylate method [63]. Nitrous oxide was analyzed on a gas chromatograph (GC 7890, Agilent Technologies) equipped with a CP-PoraPLOT Q column and a ^63^Ni electron capture detector. Isotopically labeled ammonium (^15^NH_4_^+^) was analyzed with the hypobromite oxidation assay [64,65] followed by ^15^N-N_2_ analysis on a gas chromatography-isotopic ratio mass spectrometer (GC-IRMS; VG Optima, Manchester, UK). Prior to hypobromite addition, care was taken to remove any N_2_ possibly produced during the anaerobic incubation by flushing with helium for 5 min. Headspace samples for ^15^N-N_2_O and ^15^N-N_2_ analysis were taken directly from the incubation exetainers and measured on the GC-IRMS.

### ATP analysis

Biomass-specific contents of adenosine triphosphate (ATP) of An-4 were determined using a modified protocol for ATP quantification in aquatic sediments [66]. Briefly, 1–3 pre-weighed An-4 aggregates were sonicated in 5 mL of ice-cold extractant (48 mmol L^-1^ EDTA-Na_2_ in 1 mol L^-1^ H_3_PO_4_) for 1 min and then stored on ice for 30 min. The cell suspension was centrifuged at 3000× g for 10 min and 1 mL of the supernatant was diluted 1:10 with autoclaved deionized water and adjusted to pH 7.8 with NaOH. An ATP assay mix (FLAAM, Sigma-Aldrich) and a luminometer (TD 20e Luminometer, Turner Designs) were used to quantify the extracted ATP with the firefly bioluminescence reaction. The ATP assay mix was diluted 1:25 with a dilution buffer (FLAAB, Sigma-Aldrich). Calibration standards (0–100 μmol L^-1^) were prepared from ATP disodium salt hydrate (A2383, Sigma-Aldrich) dissolved in 1:10-diluted extractant adjusted to pH 7.8. Biomass-specific ATP contents of An-4 were calculated from the ATP concentrations of the extracts and the protein contents of the An-4 aggregates.

## Competing interests

The authors declare that they have no competing interests.

## Authors’ contributions

TS, PS, TB, and DDB conceived and designed the project. SFO, AK, and PS carried out the experiments and analyzed the data. CSM and JH supplied materials and data. PS wrote the paper with help from all authors. The final manuscript was read and approved by all authors.

## Supplementary Material

Additional file 1 **Figure S1.** Time course of inorganic nitrogen species during anaerobic incubation of *A. terreus* isolate An-4. **Figure S2**. Phylogenetic position of isolate An-4 in *A. terreus*[39]. Click here for file
