# Supplementary material for: Dissimilatory nitrate reduction by Aspergillus terreus isolated from the seasonal oxygen minimum zone in the Arabian Sea
Source: BMC Microbiol. 2014 Feb 11;14:35. doi: 10.1186/1471-2180-14-35 (PMC3928326; doi:10.1186/1471-2180-14-35)
Supplement: Additional file 1 — Figure S1. Time course of inorganic nitrogen species during anaerobic incubation of A. terreus isolate An-4. Figure S2. Phylogenetic position of isolate An-4 in A. terreus[39]. [file 1471-2180-14-35-S1.doc]

**Figure S1 - Time course of inorganic nitrogen species during anaerobic incubation of *A. terreus* isolate An-4.**

This experiment was similar to the ^15^N-labeling experiment presented in the main manuscript (Experiment 2), except for the addition of ^15^NO_3_^-^ after the aerobic pre-incubation and the shorter duration of the anaerobic incubation. Note that this experiment confirmed the production of ^15^N-N_2_O from ^15^NO_3_^-^. Absolute amounts of ^15^NH_4_^+^, ^15^N-N_2_O, and ^15^N-N_2_ in the incubation vials are shown. Means ± standard deviation (n = 3).

**Figure S1**

**Figure S2 - Phylogenetic position of isolate An-4 in *A. terreus*.**

Maximum-parsimony tree based on the phylogenetic analysis of partial calmodulin sequence data of *Aspergillus* section *Terrei* [57,58]. Numbers above the branches are bootstrap values; only values above 70% are indicated. The scale bar represents the number of substitutions for a unit branch length. Phenotypic examination of isolate An-4 showed fast-growing, olive-brown colonies. Microscopic analysis showed biseriate *Aspergillus* conidiophores with subglobose conidia of 1.5-2.5 µm in diameter. The combination of these characters is in agreement with species belonging to *Aspergillus* section *Terrei* [39].

**Figure S2**
